# Clinical Signs of Radiologic Pneumonia in Under-Five Hypokalemic Diarrheal Children Admitted to an Urban Hospital in Bangladesh

**DOI:** 10.1371/journal.pone.0071911

**Published:** 2013-08-12

**Authors:** Mohammod Jobayer Chisti, Mohammed Abdus Salam, Hasan Ashraf, Abu S. G. Faruque, Pradip Kumar Bardhan, Sumon Kumar Das, K. M. Shahunja, Abu S. M. S. B. Shahid, Tahmeed Ahmed

**Affiliations:** 1 Centre for Nutrition & Food Security (CNFS), International Centre for Diarhoeal Disease Research, Bangladesh (icddr,b), Dhaka, Bangladesh; 2 Clinical Services (CS), icddr,b, Dhaka, Bangladesh; 3 Research Administration (RA), icddr,b, Dhaka, Bangladesh; University of Liverpool, United Kingdom

## Abstract

**Background:**

Clinical signs of pneumonia are often veiled in under-five diarrheal children presenting with hypokalemia, making clinical diagnosis of pneumonia very difficult in such population. However, there is no published report that describes the influences of hypokalemia on the clinical signs of pneumonia in diarrheal children. Our objective was to assess the influences of hypokalemia, and their outcome in such children.

**Methods:**

We prospectively enrolled all under-five diarrheal children (n = 180) admitted to the Special Care Ward of the Dhaka Hospital of icddr,b from September-December 2007 with radiological pneumonia who also had their serum potassium estimated. We compared the clinical features and outcome of the diarrheal children having pneumonia with (cases = 55) and without hypokalemia (controls = 125).

**Results:**

The case-fatality among the cases was 2 times higher compared to the controls, but the difference was not statistically significant (p = 0.202). In logistic regression analysis, after adjusting for potential confounders such as age of the patient, clinical dehydration, severe wasting, abnormally sleepy, lower chest wall in-drawing, nasal flaring and inability to drink on admission, under-five diarrheal children with pneumonia who presented with nutritional edema had 3 times more risk to have hypokalemia compared to those without nutritional edema (OR = 2.76, 95% CI = 1.01–7.51) and these hypokalemic children were 64% less likely to present with fast breathing (OR = 0.36, 95% CI = 0.17–0.74).

**Conclusion and significance:**

The results of our analysis are simple but may have great public health implications and underscore the importance of diligent assessment for pneumonia in under-five diarrheal children having risk of hypokalemia as in children with nutritional edema even in absence of fast breathing, a useful sign of pneumonia. This may help for early initiation of first dose of parental antibiotics along with potassium supplementation before referral to tertiary hospitals by health workers to combat probability of deaths in such population especially in resource limited settings.

## Introduction

Diarrhea and pneumonia are the two leading causes of morbidity and deaths among under-five children in developing countries [1], [2]. Among the estimated 7.6 million global deaths in under-five children in 2010, pneumonia and diarrhea accounted for 18% and 11% of the deaths respectively [3]. These two are the common co-morbidities in under-five children with high morbidity and deaths in many developing countries [1], [4]. Children with acute watery diarrhea (AWD) often also present with hypokalemia due to loss of potassium in stool [5], [6]. Clinical features of pneumonia such as difficult breathing or fast breathing may be subtle in hypokalemic diarrheal children due to muscular weakness [7]–[9], making diagnosis of pneumonia difficult in such population. Consequently, health workers in resource limited settings might miss the clinical signs of pneumonia in hypokalemic diarrheal children, delaying the initiation of appropriate antibiotics and potentially increasing the probability of deaths. It is thus very important to understand the influence of hypokalemia on the clinical features of radiological pneumonia in diarrheal children in order to develop guidelines for pneumonia diagnosis in such population and initiate appropriate management to reduce probability of deaths, especially in resource constraint settings. To our knowledge, practical importance of this issue in clinical management has not been adequately described in medical literature.

Around 80,000 under-five children attend the Dhaka Hospital of International Centre for Diarrheal Disease Research, Bangladesh (icddr,b) with diarrhea each year, and some of them also have pneumonia with concomitant hypokalemia. The aim of our study was to evaluate the influence of hypokalemia in young children with hypokalemia on the clinical features of radiological pneumonia, identify associated factors, and their outcome.

## Materials and Methods

### Ethics statement

The study (icddr,b; grant no Gr- 00233) was approved by the Ethical Review Committee (ERC) of International Centre for Diarrheal Disease Research, Bangladesh (icddr,b) and an informed verbal consent was obtained from parents or guardians of all participating children. Children whose caregivers did not give consent were not included in the study but still received standard hospital care. Originally this data has been obtained from a prospective hospital audit which was initially designed to defend the thesis in Masters of Medicine (MMed) of the primary author in the University of Melbourne (UOM), Melbourne, Australia. Although the clinical audit is routine for hospital care, and used to be done only with verbal consent from the parents or guardians of the patients following the hospital policy, parents or guardians were assured about the non-disclosure of information collected from them, and were also informed about the use of data for analysis and using the results for improving patient care activities as well as publication without disclosing the name or identity of their children. A brief verbal consent form describing the above measures to the parent or guardian was used during the audit for documentation of the consent. ERC was quite satisfied with voluntary participation, the maintenance of the rights of the participants and confidential handling of personal information by the hospital audit committee and approved this consent procedure.

### Patient enrollment

This study was performed at the Dhaka Hospital of icddr,b. Each year, the hospital provides care and treatment to over 140,000 patients of all ages. The majority of the patients come from a poor socio-economic background living in urban and peri-urban Dhaka. This being mainly a diarrhea treatment facility, essentially all patients have diarrhea with or without associated complications, but some patients, particularly young children, have co-morbidities. The majority of the patients are under-five children, and malnutrition and pneumonia are the most common co-morbidities. On arrival to the hospital, triage nurses obtain medical history and perform physical examination, and make a quick assessment of the patients, focusing on the diarrhea severity, dehydration status and associated complications, or health problems, particularly malnutrition and pneumonia. The patients are then referred to the emergency unit where physicians re-assess and admit them to an appropriate ward of the hospital. Patients with severe illnesses, including those with abnormal mental status, severe and very severe pneumonia, cyanosis, hypoxemia, suspected sepsis, and convulsions are admitted to the Special Care Ward (SCW) for further assessment, closer observation, and appropriate laboratory workup and management. After admission to the SCW, attending physicians re-evaluate the patients, initiate the needed work ups, and prescribe a management plan.

### Study design

We prospectively assessed all the children of both sex, aged 0 to 59 months, who were admitted to the SCW from September 2007 through December 2007with diarrhea and cough to diagnose radiological pneumonia. In this study, we enrolled the children who had radiological pneumonia and also had their serum potassium measured. Comparison of the clinical features of diarrhea and pneumonia was made between the children with and without hypokalemia defined as serum potassium of less than 3.5 mMol/L [10]. Pneumonia was diagnosed based on radiological evidence of consolidation or patchy opacities [11]. Diarrhea was defined as the passage of three or more abnormally loose or watery stool in the previous 24 hours [12]. Relevant clinical information was collected soon after their enrollment into the study, after obtaining verbal consents from parents/attending caregivers by the attending physician.

Standard hospital guidelines were followed in the clinical management of the study children, which included correction of dehydration using either oral rehydration salt solution (for those with some dehydration) and/or intravenous fluids (for those with severe dehydration and also those who were unable to drink due to any reason), as appropriate; antimicrobial therapy, when indicated; feeding, and administration of micronutrients (vitamins and minerals) when indicated. Management of severe protein-energy malnutrition was done in accordance with the hospital's protocolized guidelines [13], [14].

### Statistical Methods

We developed and pre-tested Case Report Forms for data acquisition. All data were entered onto a personal computer and edited before analysis using SPSS for Windows (version 15.0; SPSS Inc, Chicago) and Epi Info (version 6.0, USD, Stone Mountain, GA). Differences in proportions were compared by the Chi-square test and Fisher's Exact test was applied when indicated, and the differences of means were compared by Student's t-test or Mann-Whitney test, as appropriate. A probability of less than 0.05 was considered statistically significant. Strength of association was determined by calculating odds ratios (OR) and their 95% confidence intervals (CI). We performed these statistics both in univariate analyses and logistic regression. Characteristics that we analyzed included age, poor socio-economic condition [monthly income less than 5,000 taka (US$ 75)], vomiting, clinical dehydration, cough, breast-feeding status, fever (≥38°C), fast breathing (respiratory rate ≥ 50/minute in a calm child), lower chest wall in-drawing (in-drawing of the bony structures of the lower chest wall during inspiration), nasal flaring, cyanosis, grunting respiration, abnormally sleepy (in absence of dehydration or after correction), inability to drink, severe wasting [z score for weight for length/height <−3 of the WHO growth standard], nutritional edema, and outcome. Initially, we performed univariate analysis to identify characteristics that were significantly associated with hypokalemia and finally we performed logistic regression analysis of the factors that were significantly associated with hypokalemia after adjusting for potential confounders to determine the impact of hypokalemia on clinical features of pneumonia among children with diarrhea.

## Results

In total, 258 under-five diarrheal children with cough were screened and 180 of them had radiological pneumonia and were enrolled in the study, 55 (31%) and 125 of them had hypokalemia and normal serum potassium respectively. Children with hypokalemia had 2 times higher case-fatality compared to those without hypokalemia on admission, but the difference was not statistically significant (Table 1). In logistic regression analysis, after adjusting for potential confounders such as age of the patient, clinical dehydration, severe wasting, abnormally sleepy, lower chest wall in-drawing, nasal flaring and inability to drink on admission, under-five diarrheal children with radiological pneumonia who presented with nutritional edema had 3 times more risk to have hypokalemia compared to those who did not have nutritional edema and these hypokalemic children were 64% less likely to present with fast breathing (Table 2). There was no significant difference in other clinical features of pneumonia such as cough, grunting respiration, and cyanosis between the groups (Table 1). The distribution of poor socio-economic condition, vomiting, non-breast-fed status, and fever was also not different between the groups (Table 1).

**Table 1:** Clinical characteristics of under-five diarrheal children with pneumonia and hypokalemia (cases) and without hypokalemia (controls).

Characteristic	Cases	Controls	OR	95% CI	p
	(n = 55)	(n = 125)			
Age in months (median, IQR)	5.5 (3.0, 11.0)	6.0 (4.0, 12.0)	–	–	0.822
Poor socio-economic condition	28 (52)	80 (65)	0.58	0.29–1.17	0.136
Vomiting	740 (73)	79 (63)	1.55	0.74–3.31	0.283
Clinical dehydration (some/severe)	30 (56)	50 (40)	1.80	0.90–3.59	0.099
Cough	51 (93)	110 (88)	1.74	0.50–6.56	0.492
Non-breastfed	8 (15)	33 (27)	0.47	0.18–1.17	0.114
Admission Fever	50 (91)	107 (86)	1.68	0.55–5.52	0.459
Fast breathing	28 (51)	92 (77)	0.39	0.18–0.84	0.015
Lower chest wall in drawing	40 (73)	95 (77)	0.81	0.37–1.79	0.712
Nasal flaring	11 (20)	24 (19)	1.04	0.43–2.47	0.917
Grunting respiration	2 (4)	5 (4)	0.91	0.12–5.51	1.00
Cyanosis	3 (6)	10 (8)	0.66	0.14–2.77	0.757
Abnormally sleepy	29 (53)	43 (35)	2.10	1.05–4.22	0.035
Inability to drink	19 (35)	47 (38)	0.68	0.42–1.77	0.794
Nutritional edema	10 (18)	12 (10)	2.09	0.77–5.65	0.170
Severe wasting	12 (22)	25 (21)	1.08	0.06–2.52	0.999
Death	8 (15)	9 (7)	2.19	0.72–6.68	0.202

Figures represent n (%), unless specified. OR: odds ratio. CI: confidence interval.

IQR: inter-quartile range.SD: standard deviation. WHZ: weight for height z score; SpO_2 = _transcutaneously measured blood oxygen concentration.

**Table 2:** Results of logistic regression to identify factors independently associated with hypokalemia in diarrheal children with pneumonia.

Characteristics	OR	95% CI	p
Age in months	1.01	0.97–1.05	0.592
Clinical dehydration (some/severe)	1.27	0.57–2.83	0.553
Severe wasting	0.66	0.25–1.72	0.391
Nutritional edema	2.76	1.01–7.51	0.047
Abnormally sleepy	1.86	0.86–4.01	0.115
Fast breathing	0.36	0.17–0.74	0.005
Lower chest wall in-drawing	1.09	0.42–2.85	0.859
Nasal flaring	0.97	0.39–2.41	0.939
Inability to drink	0.69	0.32–1.49	0.343

## Discussion

There are two major observations of our study - diarrheal children with radiological pneumonia and hypokalemia had higher frequency of nutritional edema and were less frequently tachypnic than those without hypokalemia. Potassium is the most abundant intracellular *cation* in the human body and its homeostasis is essential for normal cellular function [15]. Derangements of potassium regulation may occur in severe malnutrition and diarrhea [7], [15]. Nutritional edema is a form of acute severe malnutrition, which is largely associated with depleted total body potassium [7]. Moreover, there may be further reduction of body potassium due to its loss in diarrheal stools [16]. Thus, the frequent observation of nutritional edema in diarrheal children with pneumonia and hypokalemia is understandable.

Our observation of less frequent fast breathing, a sign of pneumonia, in hypokalemic diarrheal children with pneumonia is a finding that has not been reported in medical literature in such population. Due to the reduced total body potassium and resultant reduced muscle power, the function of accessory muscles of respiration is likely to be compromised despite presence of pneumonia [9], [17]. Moreover, severe acute malnutrition, a strong co-factor for hypokalemia in our study population, is known for poor inflammatory response impeding appropriate elevations in the respiratory rate in children with pneumonia [9]. Therefore, our findings of less frequent fast breathing in diarrheal children with pneumonia in association with concomitant hypokalemia are comprehensible.

Our observation of nutritional edema as an independent predictor for hypokalemia and less frequent fast breathing in under-five diarrheal children with pneumonia, after adjusting for potential confounders, such as age of the patient, clinical dehydration, severe wasting, abnormally sleepy, lower chest wall in-drawing, nasal flaring and inability to drink on admission, suggests that hypokalemia in diarrheal children influences clinical signs of pneumonia by masking fast breathing. This observation is very important and has high clinical relevance in the management of diarrheal children with pneumonia, especially in resource limited settings lacking laboratory back-up to measure serum potassium. It is currently recommended that oral antibiotics be started in all children with nutritional edema and that parenteral antibiotics are started in those who appear lethargic or sickly [18]. Early identification of diarrheal children with cough, clinical features of hypokalemia, and severe malnutrition (nutritional edema) by the health workers might alert them to look for other signs of pneumonia even in absence of fast breathing in order to provide first dose of parenteral antibiotics before referring them to appropriate health hospitals for further assessment and management in an effort to reduce morbidity and deaths in such children.

We observed that diarrheal children with pneumonia and hypokalemia were more likely to be abnormally sleepy. Although the exact mechanism is not known, abnormal mentation is a feature of hypokalemia, which might be contributed by severe acute weakness [7], [19], [20]. However, this finding was significant in univariate analysis but lost level of significance in logistic regression analysis after adjustments of co-variates in the analysis. We observed higher case-fatality in diarrheal children with pneumonia and hypokalemia than those without hypokalemia; however, the difference was not statistically significant. Previous studies reported significantly higher case-fatality in diarrheal children with hypokalemia than those without hypokalemia [21], the discrepancy noted in our study might be due to small sample size, thereby, inadequate study power.

Our observation of the comparable distribution of cough, grunting respiration, and cyanosis in both the groups might be due to the fact that both the groups had the identical features of pneumonia [22] or these are the results of overmatching at the time analysis and do not have any known patho-physiological relation with hypokalemia. The failure to find the association of poor socio-economic condition, vomiting, non-breast-fed status, and fever among the groups might also be due to same reason or might be due to homogeneity of our study population.

Small sample size is the main limitation of our study, which might have impacted level of statistical significance for some of the variables among the groups.

Based on the findings, we may conclude that case-fatality may be expected to be higher in under-five diarrheal children with pneumonia who also have hypokalemia and thus such children should receive closer observations at health care facilities. Under-five diarrheal children with pneumonia, who also have nutritional edema, are at higher risk of hypokalemia and they are less likely to have fast breathing. Thus, in addition to potassium supplementation, parenteral antibiotics therapy should be initiated in all children with nutritional edema and diarrhea who also have cough including those without fast breathing before referral to tertiary hospital by the health workers and this may help to combat probability of deaths in such population with cough especially in resource poor settings. Further researches with larger sample size may be conducted to confirm or refute our observations.
